# Control Interactions in the Theory of Planned Behavior: Rethinking the Role of Subjective Norm

**DOI:** 10.5964/ejop.v16i3.2056

**Published:** 2020-08-31

**Authors:** Francesco La Barbera, Icek Ajzen

**Affiliations:** a Department of Political Sciences, University of Naples Federico II, Naples, Italy; b Department of Psychological and Brain Sciences, University of Massachusetts Amherst, Amherst, MA, USA; ZPID – Leibniz Institute for Psychology Information, Trier, Germany

**Keywords:** TPB, perceived behavioral control, attitude, subjective norm, interaction effects, moderation

## Abstract

Research with the theory of planned behavior (TPB) has typically treated attitude (ATT), subjective norm (SN), and perceived behavioral control (PBC) as independent predictors of intention (INT). However, theoretically, PBC moderates the effects of ATT and SN on intention. In three studies dealing with different behaviors (voting, reducing household waste, and energy consumption) we show that greater PBC tends to strengthen the relative importance of ATT in the prediction of intention, whereas it tends to weaken the relative importance of SN. The latter pattern was observed in relation to injunctive as well as descriptive subjective norms, and it may help explain the relatively weak relation between SN and INT frequently observed in TPB studies.

Following publication of the seminal book on attitude research by Fishbein and Ajzen (1975), the theory of reasoned action (TRA) became a leading framework for psychological explanations of human behavior (Ajzen & Fishbein, 1980; see Sheppard, Hartwick, & Warshaw, 1988). The TRA posited that the immediate antecedent of a particular behavior is the intention (INT) to perform the behavior in question. Intention, in turn, was said to be determined by attitude toward the behavior (ATT)—the individual’s favorable or unfavorable evaluation of the behavior—and subjective norm (SN), the perceived social pressure to perform or not to perform the behavior.

A major limitation of the TRA was the requirement that the behavior under consideration be under volitional control (Fishbein & Ajzen, 1975), a requirement that greatly limited the theory’s applicability. To enable prediction and explanation of behavior over which control is incomplete, Ajzen (1985, 1991) reformulated the TRA by adding perceived and actual behavioral control to the model, renaming it the theory of planned behavior (TPB). In the TPB, intentions are posited to predict behavior to the extent that the actor is capable of performing the behavior, i.e., to the extent that *actual* control over behavioral performance is high. In the relatively few studies that have examined this proposition, perceived behavioral control (PBC) has been used as a proxy for actual control, with mixed results (see Yang-Wallentin et al., 2004 for a review). Perhaps less know, PBC is also said to moderate the effects of ATT and SN on behavioral intentions (see Ajzen, 1985, 2002). Favorable attitudes and subjective norms should lead to the formation of a favorable intention only to the extent that people also believe that they are capable of carrying out the behavior, i.e., have high perceived control over the behavior, or a high level of perceived self-efficacy (Bandura, 1997).

Despite the large number of studies stimulated by the TPB over the years, this interaction hypothesis has received little attention (Yzer & van den Putte, 2014). Investigators have generally tested the simpler additive model in which INT is predicted from ATT, SN, and PBC (Ajzen, 2002). Although the postulated moderating effects of PBC are intuitively compelling and grounded in theory, studies examining them have rarely obtained empirical support. This failure may in large part be due to methodological difficulties (Ajzen, 2002; Fishbein & Ajzen, 2010; Yang-Wallentin et al. 2004; Yzer & van den Putte, 2014). One major problem is that the statistical power of interaction tests is very sensitive to the distribution of the predictor and moderator variables. As Ajzen (2002, p. 667) has noted, “Logically, perceived behavioral control, rather than having a direct effect, is expected to interact with attitudes and with subjective norms in determining intentions, and with intentions in its effects on behavior. Empirically, however, interactions of this kind can be expected only if values of the predictor variables cover the full range of possible scores, such that the product term is fully expressed in the prediction” (see Ajzen & Fishbein, 2008 for evidence in support of this argument).

Unfortunately, the data collected in TPB studies often show floor or ceiling effects with accompanying low variance in at least one of the measured variables – a circumstance that poses a major challenge for research on interactions (Yzer & van den Putte, 2014). Researchers interested in testing interactions involving PBC must make sure that their measures of ATT, SN, and PBC cover as much of the full range as possible and screen their data for excess skewness and kurtosis. In addition, there should also be sufficient variance in the dependent intention measure. Avoiding these methodological problems usually involves choice of target behaviors that meet the necessary prerequisites, safeguards that are rarely evident in TPB research.

## Aims and Hypotheses

In this article we report the results of three studies that tested the hypothesized interactions between attitude and perceived behavioral control (ATT x PBC) and between subjective norm and perceived behavioral control (SN x PBC) in the prediction of intentions. Replications of this kind are needed in light of the “replicability crisis” in psychological science (Nosek et al., 2015) and to generalize findings across behaviors that may vary in terms of meeting the prerequisites for testing interaction effects.

Several investigators (Conner & McMillan, 1999; Hukkelberg et al., 2014; Kothe & Mullan, 2015; Yzer & van den Putte, 2014) have reported a significant interaction between ATT and PBC in the prediction of intention. In line with expectations, the higher the perceived control over the behavior, the stronger was the association between attitude and intention. In a multiple regression analysis, this interaction effect was evident in a significant *positive* regression coefficient for the ATT x PBC term. We expected to find a similar result in our studies.

Empirical findings regarding the SN x PBC interaction have been inconsistent. Of the few studies that tested this interaction, some found a non-significant effect of PBC on the prediction of intention from SN (Earle et al., 2020; Kothe & Mullan, 2015; Umeh & Patel, 2004); at least one study (Yzer & van den Putte, 2014) reported a positive effect, such that the prediction of intention from SN was *better* under high as compared to low PBC; yet another study (Castanier et al., 2013) found a significant *negative* SN x PBC interaction coefficient, indicating that the prediction of intention from SN was *weaker* under high as opposed to low PBC. Castanier et al. concluded that the more people felt control over performing a behavior (in their research, drinking and driving or disobeying road signs), the less they were influenced by peer pressure in forming their intentions.

Although perhaps unexpected for researchers in this field, the negative effect of PBC on the prediction of intention from SN is not unreasonable in light of past theory and research in social psychology. Several classical experiments on conformity (e.g., Sherif & Harvey, 1952; see also Latané & Darley, 1968) have shown that degree of conformity increases as the behavioral task becomes more ambiguous or difficult—a situation that challenges the individual’s sense of mastery. Research on self-efficacy (e.g., Bandura, 1982) also suggests that perceived control is positively associated with autonomy and negatively associated with susceptibility to social pressure. As noted by Jones (1986, p. 267), “individuals with low levels of self-efficacy may more readily accept definitions of situations offered by others.” Finally, in a study guided by the TPB, Hill et al. (1996) concluded that the influence of subjective norms may be especially important for novel behaviors that are challenging in terms of control. Taken together, these considerations led us to hypothesize that the regression coefficient of SN in the prediction of intention *declines* as the level of PBC increases.

## Study 1

### Overview

In Study 1 we tested the additive and interactive effects of ATT, SN and PBC in the prediction of voting intentions. Specifically, as part of a research program on people’s views regarding the European Union (EU), we collected data on intentions to vote in favor of EU integration, and we also assessed ATT, SN, and PBC with respect to this behavior. In addition, we included a measure of motivation to comply with the expectations of eight significant others identified in a pilot study. Our hypothesis regarding the moderating effect of PBC on the SN-INT relation is based on the idea that individuals high in perceived behavioral control are less influenced by social norms than are individuals low in PBC. This implies that motivation to comply with significant others should be negatively correlated with perceived behavioral control.

### Materials and Method

#### Participants

Four hundred and two participants (216 females, aged 18 to 82, *M*_age_ = 37.22, *SD*_age_ = 14.04), recruited in four public buildings in Italy, completed the scales described below. The survey was conducted in Italian (the items described in this paper are translations from the Italian).

#### Measures

##### Intention

To assess participants’ intentions to vote in favor of EU integration, we used five items (e.g., “I would vote for EU to become a single country”) based on previous studies conducted on the topic (La Barbera, 2015; La Barbera, Cariota Ferrara, & Boza, 2014). Answers were collected on 7-point scales ranging from “*definitely not*” to “*yes, definitely*,” and then averaged across items to produce a single composite score, with higher values indicating a more favorable intention to vote for EU integration (Cronbach’s α = .85).

##### Attitude

Attitudes toward voting in favor of EU integration were assessed by asking participants to rate “For me, voting in favor of European integration is:” on five 7-point bipolar adjective scales (e.g. “*good* – *bad*”, “*unpleasant* – *pleasant*”). Responses were aggregated into a composite measure by averaging the scores on the five scales (Cronbach’s α = .84). Higher values indicate more positive attitudes.

##### Subjective norm

Four items were used to measure subjective norm (e.g.: “Most people who are important to me believe that I should/I should not vote in favor of European integration”) Participants answered on 7-point scales. The answers were again aggregated into a single score by computing the mean across the scales (Cronbach’s α = .67). Higher values indicate greater social support for voting in favor of European integration.

##### Perceived behavioral control

Four items were used to measure perceived behavioral control (e.g.: “Whether I will vote in favor of European integration depends exclusively on me” (Cronbach’s α = .64). Responses on the 7-point scales were averaged. Higher values indicate higher perceived control.

##### Motivation to comply

Eight items were used to measure the individual’s motivation to comply with significant referents emerged in a pilot study (e.g.: “Generally speaking, how much do you care about what your friends think you should do?”). Participants answered on 7-point scales ranging from “*not at all*” to “*very much*.” The answers were aggregated into a single score by computing the mean across the scales (Cronbach’s α = .86). Higher values indicate higher motivation to comply.

### Results and Discussion

As noted, distribution is a major issue when studying interactions. We first inspected the range of scores for the variables involved in moderation tests. The requirement that these scores cover the full range of the 7-point scale were met. They ranged from 1 to 7 for ATT and SN, and from 1.5 to 7 for PBC. Intention scores also covered the full range. Second, it can be seen in Table 1 that the sample mean scores of all variables were close to the scale’s midpoint of 4.0. Finally, we assessed the shape of the study variables’ frequency distributions. As a conventional rule of thumb, values of skewness and kurtosis should not exceed the threshold of ± 2 (Field, 2009; Gravetter & Wallnau, 2014). For the variables assessed in our study, standard deviations exceeded 1.0, skewness ranged from -.166 to .312, and kurtosis ranged from -.030 to -.758. Taken together, these findings suggest that our measures met all requirements for testing the hypothesized interactions.

**Table 1:** Correlations, Means, and Standard Deviations of Study Variables: Study 1

Variable	1	2	3	4	5
1. INT	4.24 (1.58)				
2. ATT	.302***	4.75 (1.35)			
3. SN	.252***	.552***	4.57 (1.14)		
4. PBC	.249***	.521***	.422***	4.99 (1.25)	
5. MC	.012	-.093	-.091	-.192***	2.80 (1.28)

*Note.* The table shows Pearson’s *r* correlation coefficients. Diagonal cells report the variable means (standard deviations in parentheses). INT = Intention. ATT = Attitude. SN = Subjective norm. PBC = Perceived behavioral control. MC = Motivation to comply.

***p < .001.

Correlations, means, and standard deviations of the study variables are shown in Table 1.

As expected, the TPB constructs were significantly intercorrelated. Interestingly, PBC was negatively correlated with motivation to comply. Next, we used a hierarchical regression analysis to predict intentions from ATT, SN, and PBC (Step 1), followed on the second step by the two hypothesized interactions. The variables were mean-centered before calculating the interaction terms. Results are summarized in Table 2.

**Table 2:** Hierarchical Regression Analysis of the Intention to Vote for EU Integration: Study 1

Predictor	*b*	95% CI	*t*
Step 1 (*R*^2^ = .114***)			
ATT	0.220**	[0.07, 0.37]	2.89
SN	0.149	[-0.02, 0.31]	1.76
PBC	0.156*	[0.01, 0.30]	2.08
Step 2 (*R*^2^ = .134***; Δ*R*^2^ = .021*)			
ATT	0.217**	[0.07, 0.37]	2.90
SN	0.187*	[0.02, 0.36]	2.17
PBC	0.171*	[0.03, 0.32]	2.30
ATT x PBC	0.099*	[0.01, 0.19]	2.04
SN x PBC	-0.169**	[-0.29, -0.04]	-2.69

*Note.* Unstandardized regression coefficients are reported. CI = Confidence interval. INT = Intention. ATT = Attitude. SN = Subjective norm. PBC = Perceived behavioral control.

*p < .05. **p < .01. ***p < .001.

It can be seen that ATT and PBC were significantly associated with intention, whereas SN was not. On the second step, both hypothesized interactions proved significant, raising explained variance by 2.1%, *F*(2, 357) = 4.29, *p* = .014. However, the regression coefficients of ATT and SN had opposite signs, suggesting a different interaction pattern. We conducted a simple slope analysis to clarify the nature of the two interactions.

**Figure 1:**
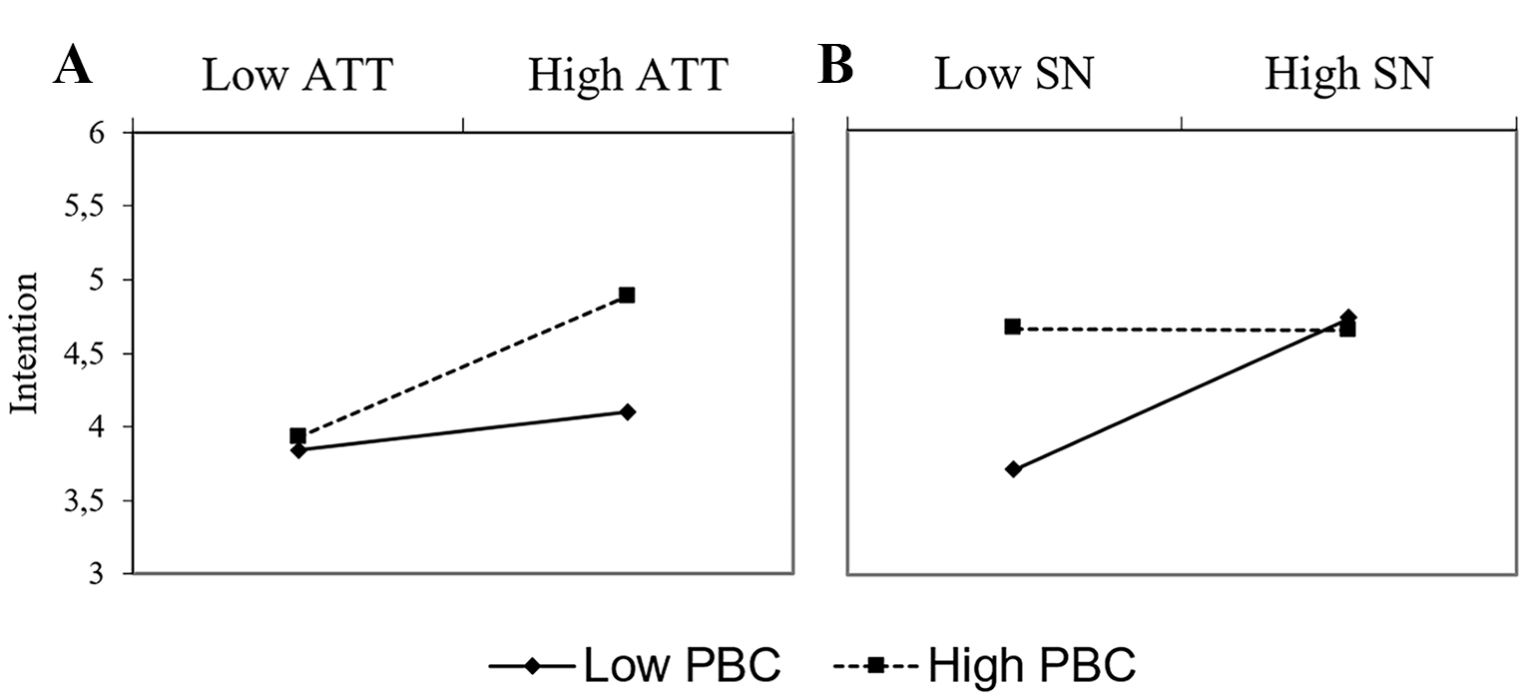
Simple slope analysis of the ATT x PBC (A) and SN x PBC (B) interaction: Study 1.

The interaction between attitude and perceived behavioral control showed a pattern in line with our hypothesis and previous research. As can be seen in Figure 1A, the relation between attitude and intention was stronger when PBC was high than when it was low. In fact, when PBC was 1*SD* below the mean the ATT-INT relation was not significant (*b* = 0.09, *t* < 1), whereas it proved statistically significant when PBC was 1*SD* above the mean (*b* = 0.35, *t* = 3.44, *p* < .001).

The simple slope analysis also elucidates the contrary pattern of interaction between subjective norms and perceived behavioral control. When PBC was high, intention to vote in favor of EU integration remained relatively stable at the different levels of perceived social pressure. In fact, when PBC was 1*SD* above the mean the relation between subjective norm and intention was not statistically significant (*b* = 0.03, *t* < 1). By contrast, when PBC was below the mean, there was a significant positive relation between SN and INT (*b* = 0.40, *t* = 3.12, *p* < .01).

Consistent with our hypotheses, the results of our first study suggest that in forming their intentions, participants rely on social pressure primarily when their sense of control is low rather than high. In other words, they seem to be more motivated to comply with the perceived normative expectations and behaviors of important social referents only when PBC is relatively low. To assess the validity of this interpretation, participants’ PBC was split at the median, creating a dichotomous variable with 0 (below median) representing low PBC, and 1 (above median) representing high PBC. In line with our interpretation, we found that motivation to comply with significant referents was higher for participants with low PBC, *M* = 3.01, *SD* = 1.27, compared to participants with high PBC, *M* = 2.58, *SD* = 1.24, *t*(385) = 3.33, *p* < .001. This result is in line with the significant and negative bivariate correlation found between motivation to comply and perceived behavioral control (see Table 1). Overall, these findings support our hypothesis regarding the prediction of intention from SN as moderated by PBC.

## Study 2

### Overview

In Study 2, we replicated the first study using a different target behavior, namely reducing household food waste. In recent years, the topic of food waste reduction has been receiving a great deal of attention in scientific and public debates. This is primarily due to its negative impact on the environment (Amato et al., 2019; Del Giudice et al., 2016; La Barbera, Del Giudice, & Sannino, 2014). Recently, several articles have reported studies that addressed the issue in the framework of the TPB (e.g., La Barbera et al., 2016; Riverso et al., 2017; Stancu et al., 2016; Stefan et al., 2013). Our second study went beyond these past efforts to examine the proposed moderating effects of perceived behavioral control, in relation to the prediction of intentions to reduce household food waste from attitudes and subjective norms.

### Materials and Method

#### Participants

A total of 300 questionnaires were distributed in three public buildings of a metropolitan area in Italy. The data of 18 questionnaires were dropped because participants failed to answer all questions. The final sample consisted of 282 participants (178 females), with age ranging from 18 to 76 (*M* = 33.44, *SD* = 12.64) who declared to be involved in cooking and shopping food at home. The survey was conducted in Italian (the items described in this article are translations from the Italian). It was adapted from Stefan et al. (2013), who conducted a previous TPB study on reducing household food waste.

#### Measures

##### Intention

Two items were used to measure participants’ intentions to not throw away food: “I intend not to throw away food” and “In general, I try very hard not to throw away food.” Answers were collected on 7-point scales and then averaged across items to produce a single composite score, with higher values indicating a stronger intention not to throw away food (Spearman-Brown Rho = .77).

##### Attitude

Attitudes toward throwing food away were measured by three items (e.g.: “Throwing away food does not bother me” – reverse coded). Participants responded on 7-point scales ranging from “*strongly disagree*” to “*strongly agree*.” Responses were aggregated into a composite measure by averaging the scores on the three scales (Cronbach’s α = .55). Higher values indicate more negative attitudes towards throwing away food.

##### Subjective norm

Two items were used to measure subjective norms: “Most people important to me disapprove of me cooking/preparing more than enough food” and “Most people important to me disapprove of me throwing food away.” Participants answered on 7-point scales ranging from “*strongly disagree*” to “*strongly agree*.” The answers were again aggregated into a single score by computing the mean across the two scales (Spearman-Brown Rho = .62). Higher values indicate greater social support for reducing food waste.

##### Perceived behavioral control

Three items were used to measure perceived behavioral control (e.g., “I am able to buy exactly the amount of food that my household needs”). Responses on the 7-point scales (from “*strongly disagree*” to “*strongly agree*”) were averaged (Cronbach’s α = .75). Higher values indicate higher perceived control over avoiding food waste.

### Results and Discussion

Basic descriptive statistics of the study variables are provided in Table 3, together with bivariate correlations. The scores of all study variables covered the full range on the 7-point scales, from 1 to 7. However, in contrast to the first study, the means tended to be positive except for PBC, which had a mean score close to the scale’s midpoint. Skewness and kurtosis were within the thresholds of ± 2 for all variables. Overall, then, the distributions of our study variables met the requirements for testing the hypothesized interactions.

**Table 3:** Correlations, Means and Standard Deviations of Study Variables: Study 2

Variable	1	2	3	4
1. INT	5.58 (1.48)			
2. ATT	.154**	5.69 (1.29)		
3. SN	.363***	.055	5.02 (1.55)	
4. PBC	.413***	.181**	.281***	4.45 (1.32)

*Note.* The table shows Pearson’s *r* correlation coefficients. Diagonal cells report the variable mean (standard deviation in parentheses). INT = Intention. ATT = Attitude. SN = Subjective norm. PBC = Perceived behavioral control.

**p < .01. ***p < .001.

We again used a hierarchical regression analysis for assessing the significance and size of the effects of the three TPB factors and their hypothesized interactions. On the first step, intention was regressed on attitude, subjective norm and perceived behavioral control. On the second step the two-way interactions (ATT x PBC and SN x PBC) were entered as predictors of intention. The variables were mean-centered before calculating the interaction terms. Results are summarized in Table 4.

**Table 4:** Hierarchical Regression Analysis of the Intention to not Throw Food Away: Study 2

Predictor	*b*	95% CI	*t*
Step 1 (*R*^2^ = .254***)			
ATT	0.093	[-0.03, 0.21]	1.53
SN	0.256***	[0.15, 0.36]	4.92
PBC	0.334***	[0.22, 0.45]	5.85
Step 2 (*R*^2^ = .288***; Δ*R*^2^ = .034**)			
ATT	0.112	[-0.01, 0.23]	1.85
SN	0.258***	[0.16, 0.36]	5.03
PBC	0.305***	[0.19, 0.42]	5.86
ATT x PBC	-0.04	[-0.14, 0.05]	< 1.00
SN x PBC	-0.111**	[-0.18, -0.04]	-3.05

*Note.* Unstandardized regression coefficients are reported. CI = Confidence interval.

**p < .01. ***p < .001.

**Figure 2:**
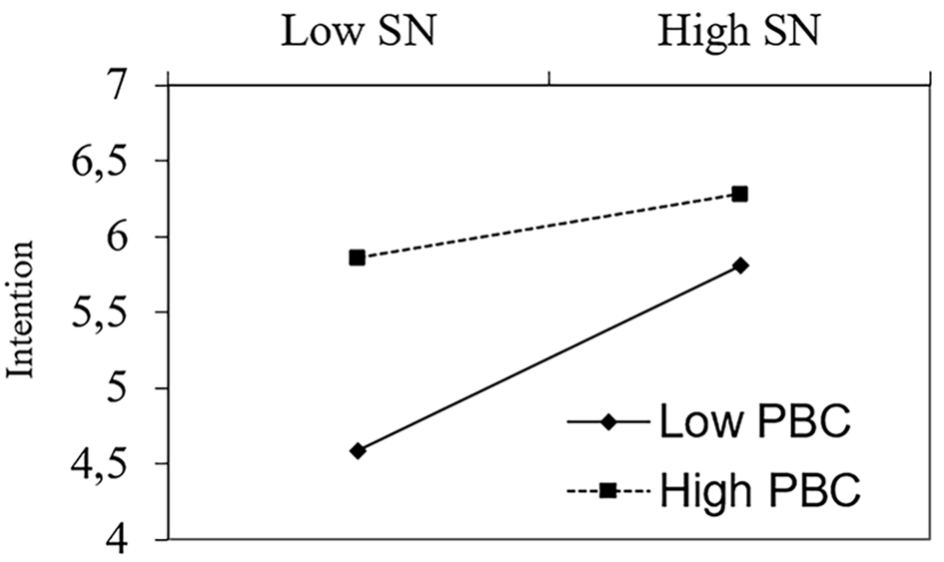
Simple slope analysis of the SN x PBC interaction: Study 2.

It can be seen that SN and PBC significantly predicted intention, whereas ATT did not. Adding the interaction terms on step 2 increased explained variance by 3.4%, *F*(2, 275) = 6.61, *p* = .002, but only the interaction between SN and PBC proved statistically significant. A simple slope analysis (see Figure 2) showed that when PBC was 1*SD* above the mean, the SN-INT relation was non-significant (*b* = 0.08, *t* = 1.14, *p* = .25), whereas it was statistically significant when PBC was 1*SD* below (*b* = 0.38, *t* = 5.53, *p* < .001, respectively). In sum, the results with respect to the SN x PBC interaction showed a pattern similar to the results of Study 1: only when PBC was low did subjective norm significantly predict intention to not throw away food. However, contrary to the results of the first study, which dealt with voting, intentions to not throw away food were unrelated to attitude toward this behavior and the interaction between attitude and perceived behavioral control was not significant.

## Study 3

### Overview

In Study 3, we further tested our interaction hypotheses on a different behavioral intention. In addition, we compared the moderating role of PBC with respect to two different kinds of subjective norms, namely injunctive and descriptive norms (Cialdini & Trost, 1998; Fishbein & Ajzen, 2010). Injunctive subjective norms refer to the perceived behavioral expectations of important social referents, whereas descriptive subjective norms refer to whether these referents are themselves perceived to perform the behavior. We aimed to explore whether the particular interaction pattern between SN and PBC found in the previous two studies would be found in relation to each of these two different kinds of norms. In order to pursue these goals, we addressed the intention to reduce individual energy consumption, which is a pro-environmental behavior (PEB). Previous research on PEB has often used the TPB framework with good results in terms of predictive validity (e.g., de Leeuw et al., 2015).

### Materials and Method

#### Participants

One hundred and fifty-eight university students (148 females, aged 18 to 24, *M*_age_ = 19.25, *SD* = 1.09) completed a web-administered questionnaire during a class session, containing the scales described below. The survey was conducted in Italian (the items described are translations from the Italian).

#### Measures

##### Intention

Three items were used to assess intentions to reduce energy consumption (e.g., “Overall, I intend to reduce my energy consumption”). Answers were collected on 7-point scales and then averaged across items to produce a single composite score, with higher values indicating intention not to throw food away (Cronbach’s α = .75).

##### Attitude

Attitudes was assessed by asking participants to rate “For me, reducing my energy consumption is:” on two 7-point bipolar adjective scales (“*unpleasant* – *pleasant*” and “*not useful* – *useful*”). Responses were aggregated into a composite measure by averaging the scores on the two scales (Spearman-Brown Rho = .67). Higher values indicate more positive attitudes.

##### Subjective norm – injunctive (SNi)

Three items were used to measure injunctive norms (e.g., “Most people who are important to me believe that I should reduce my energy consumption”). Participants responded on 7-point scales ranging from “*strongly disagree*” to “*strongly agree*.” The answers were aggregated into a single average score (Cronbach’s α = .58). Higher values indicate greater perceived social pressure in favor of reducing energy consumption.

##### Subjective norm – descriptive (SNd)

Three items were used to measure descriptive norms: (e.g., “Most students try to reduce their energy consumption”). Participants answered on 7-point scales (“*strongly disagree*” to “*strongly agree*”). The answers were aggregated into a single average score (Cronbach’s α = .76). Higher values indicate descriptive norms in favor of reducing energy consumption.

##### Perceived behavioral control

Two items were used to measure perceived behavioral control: “I am able reduce my energy consumption” and “I feel capable of reducing my energy consumption.” Responses on the 7-point “*strongly disagree*” to “*strongly agree*” scales were averaged (Spearman-Brown Rho = .85). Higher values indicate higher perceived control.

### Results and Discussion

Descriptive statistics of the study variables are provided in Table 5, together with bivariate correlations. The TPB constructs were significantly intercorrelated, except for the non- significant correlation of descriptive norms with injunctive norms and with perceived behavioral control. Scores on all variables covered the full range, from 1 to 7, with means close to the midpoint except for PBC, which was about one scale point above the midpoint. Importantly, for all study variables, skewness and kurtosis were under the threshold of ± 2, with skewness ranging from -.678 to .202, and kurtosis ranging from -.368 to .315. Overall, then, the distributions of scores on all study variables met the requirements for testing interaction hypotheses.

**Table 5:** Correlations, Means and Standard Deviations of Study Variables’ Aggregate Scores: Study 3

Variable	1	2	3	4	5
1. INT	4.59 (1.41)				
2. ATT	.694***	4.45 (1.26)			
3. SNi	.424***	.224**	4.75 (1.24)		
4. SNd	.254**	.268**	-.037	3.32 (1.24)	
5. PBC	.526***	.563***	.281***	.078	5.06 (1.41)

*Note.* The table shows Pearson’s *r* correlation coefficients. Diagonal cells report the variable mean (standard deviation in parentheses). INT = Intention. ATT = Attitude. SNi = Subjective norm (injunctive). SNd = Subjective norm (descriptive). PBC = Perceived behavioral control.

**p < .01. ***p < .001.

Hierarchical regression analysis was used for assessing the prediction of intentions from ATT, SNi, SNd, and PBC and of the hypothesized interactions. On the first step, intention was regressed on ATT, SNi, SNd, and PBC, and on the second step three two-way interactions were entered in the model, namely ATT x PBC, SNi x PBC, and SNd x PBC. The measures were mean-centered before calculating the interaction terms. Results are provided in Table 6.

**Table 6:** Stepwise Regression Analysis of the Intention to Reduce Individual Energy Consumption: Study 3

Predictor	*b*	95% CI	*t*
Step 1 (*R*^2^ = .585***)			
ATT	0.574***	[0.43, 0.72]	7.78
SNi	0.309***	[0.19, 0.43]	5.02
SNd	0.129*	[0.01, 0.25]	2.09
PBC	0.157*	[0.03, 0.28]	2.47
Step 2 (*R*^2^ = .621***; Δ*R*^2^ = .037**)			
ATT	0.502***	[0.35, 0.65]	6.76
SNi	0.305***	[0.19, 0.42]	5.10
SNd	0.119*	[0.00, 0.24]	1.98
PBC	0.221**	[0.90, 0.35]	3.32
ATT x PBC	0.104*	[0.20, 0.18]	2.60
SNi x PBC	-0.117**	[-0.19, -0.04]	-3.07
SNd x PBC	-0.108*	[0.19, 0.02]	-2.47

*Note.* Unstandardized regression coefficients are reported. CI = Confidence interval. INT = Intention. ATT = Attitude. SNi = Subjective norm (injunctive). SNd = Subjective norm (descriptive). PBC = Perceived behavioral control.

*p < .05. **p < .01. ***p < .001.

**Figure 3:**
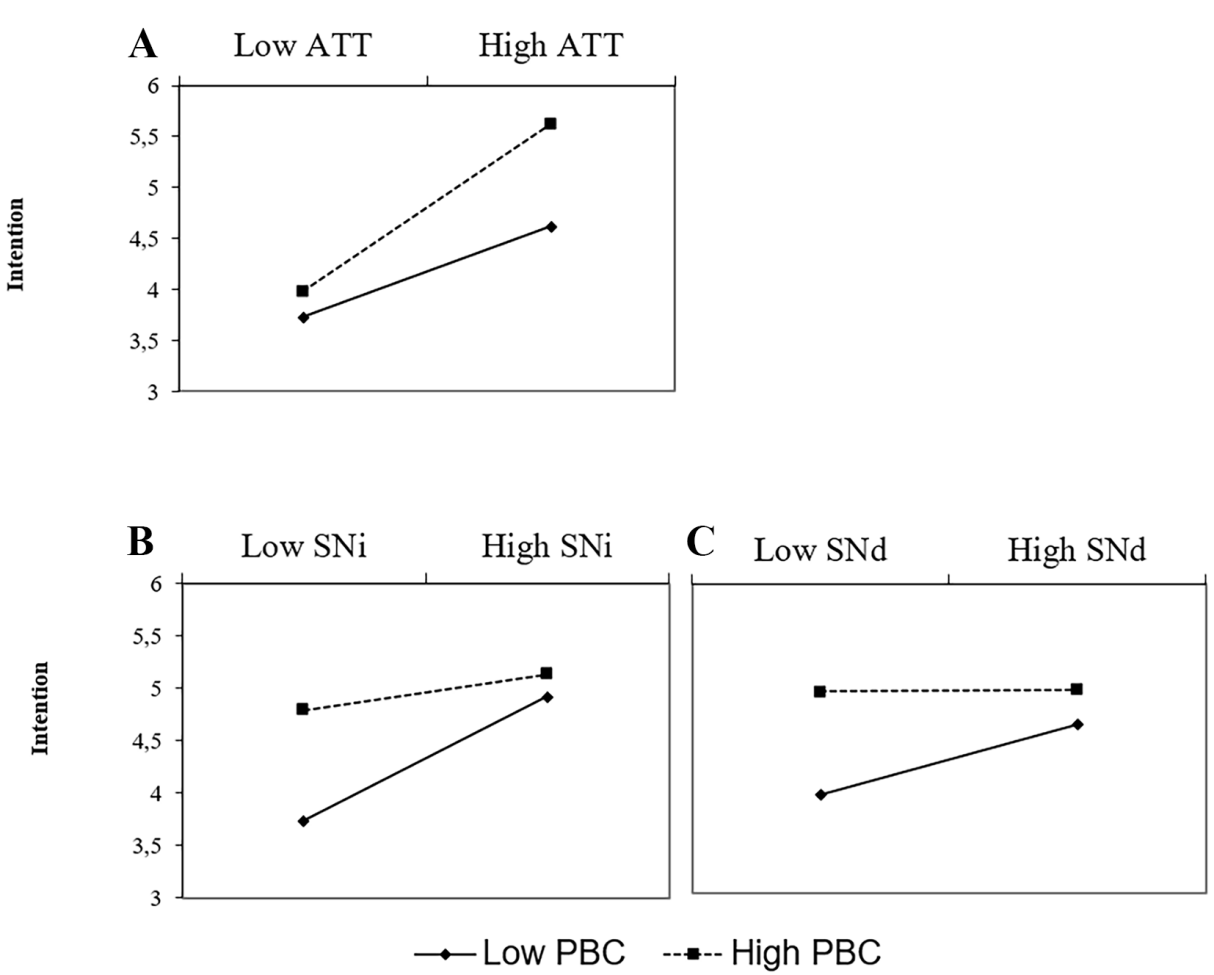
Simple slope analysis of the ATT x PBC (A), SNi x PBC (B), and SNd x PBC (C) interactions: Study 3.

It can be seen that all of the four TPB constructs significantly predicted intention to conserve energy. The model accounted for a substantive proportion of variance, *R*^2^ = .585. After entering the interaction terms on the second step, the model accounted for an additional 3.7% of the variance in intentions, *F*(3, 150) = 4.86, *p* = .003. All three interactions proved statistically significant. As in Study 1, the interactions of PBC with attitude and the two norms had opposite signs. To explore the nature of these interactions, we again conducted a simple slope analysis (see Figure 3).

The simple slope analysis showed that the ATT-INT relation was significant at different levels of PBC, but the strength of the relation increased as a function of PBC (see Figure 3A). When PBC was 1*SD* below the mean, the relation was much weaker (*b* = 0.35, *t* = 3.37, *p* < .001) than when PBC was 1*SD* above the mean (*b* = 0.65, *t* = 8.01, *p* < .001).

The relation between injunctive subjective norms and intentions was not significant when PBC was 1*SD* above the mean (*b* = 0.14, *t* = 1.70, *p* = .09), but it was statistically significant when PBC was 1*SD* below the mean (*b* = 0.47, *t* = 5.92, *p* < .001). Furthermore, the results showed the same pattern for the relation between descriptive norms and intentions: when PBC was 1*SD* above the mean the SNd-INT relation was not significant (*b* = 0.03, *t* < 1) whereas it was statistically significant when PBC was 1*SD* below the mean (*b* = 0.27, *t* = 3.32, *p* = .001). These findings replicate the results of the previous two studies in relation to injunctive subjective norms and generalize them to descriptive subjective norms.

## General Discussion and Conclusion

From a theoretical perspective, perceived behavioral control is expected to moderate the effects of attitude toward the behavior and of subjective norm on intention, and the effect of intention on behavior. However, although the theory of planned behavior has stimulated a great deal of research, tests of these interaction hypotheses have rarely been reported. This could be due to the absence of strong empirical support for these interaction effects in early research inspired by the TPB. The studies reported in the present article demonstrate the important moderating role of perceived behavioral control in the prediction of intention from attitude and subjective norms, suggesting that future research based on the TPB should pay renewed attention to interactions effects.

In line with previous research (Hukkelberg et al., 2014; Yzer & van den Putte, 2014), Studies 1 and 3 supported the hypothesis that PBC moderates the extent to which attitudes predict intentions. In study 2, intentions to avoid food waste were unrelated to attitude, and the interaction between attitude and perceived behavioral control was not significant. These unexpected findings may be due to the fact that our measure of attitude in the second study was less reliable (Cronbach’s alpha = .55) than in Studies 1 and 3 (Cronbach’s alpha = .89 and .67, respectively). The results of Study 2 regarding the lack of an interaction between ATT and PBC must therefore be interpreted with caution. Overall, however, and in line with the relatively few studies already existing on the topic, the current research supports the idea that the predictive power of attitude in relation to intention increases with PBC. In some cases, as in our first study, the relation between attitude and intention may not even be significant when PBC is low.

As to the SN x PBC interaction, all three studies showed it as statistically significant. Simple slope analyses revealed a pattern of relations opposite to the one involving attitudes and perceived behavioral control. In all three studies, subjective norms predicted intention better when perceived behavioral control was low rather than high. This pattern was found in relation to different populations and behaviors, injunctive as well as descriptive subjective norms, and slightly different measures of the TPB constructs. These findings provide early yet robust support for a pattern of interaction between perceived social pressure and perceived behavioral control that differs from the pattern for the ATT x PBC interaction. Whereas greater perceived behavioral control tends to *increase* the relative importance of attitude in the prediction of intention, it tends to *decrease* the importance of subjective norm.

As we noted in the introduction, results of previous studies on interaction effects in the TPB have been inconsistent. Several studies found a non-significant SN x INT interaction. These studies, however, failed to carefully consider the distribution of variables that are hypothesized to interact, a shortcoming that was remedied in our studies. Another reason for the different results may be related to the fact that although the three studies reported in this article replicated results across three different behaviors, all three were behaviors collective in nature. To be sure, voting for European integration, reducing household food waste, and lowering energy consumption are behaviors that are performed by individuals, but attainment of their goals (passage of a referendum favoring greater EU integration, a significant reduction of food waste, and lower overall energy consumption) also depend on the behaviors of others. In contrast, in the study by Yzer and van den Putte (2014), which found that the relative importance of subjective norm increased as a positive function of PBC, the dependent variable was intention to quit smoking, arguably an individualistic behavior that does not depend on the actions of others. Similarly, two studies that found a non-significant interaction between SN and INT (Earle et al., 2020; Kothe & Mullan, 2015) were also concerned with individualistic behaviors. The contrast between collectivistic and individualistic behaviors may explain why our results differed from those of previous studies. More research with individualistic behaviors is needed to confirm this conjecture. It is up to future research to explore whether the nature of the behavior (collectivistic, individualistic, addictive, health-related, consumer behavior, etc.) affects the pattern of interactions between SN and PBC.

It is worth noting that in many TPB studies, subjective norms tend to have a relatively weak or nonsignificant regression coefficient in the prediction of intention (e.g., Mahon, Cowan, & McCarthy, 2006; White et al., 2008; see also the meta-analytic review by Armitage & Conner, 2001), leading to the conclusion that subjective norms are of little importance in determining behavioral intentions. The significant SN x PBC interactions revealed in the present studies suggest that this conclusion may be unfounded. Our studies indicate that such results could be due to the neglect of the SN x PBC interaction term in the statistical model. Thus, in our first study, SN had a non-significant regression coefficient in the prediction of intention, but our hierarchical regression analysis revealed a significant interaction with PBC, showing that prediction of intention from subjective norm was significant for participants with relatively low PBC. These findings draw attention to the important role subjective norms may play in the prediction of intention despite their frequently weak main effects.

With respect to research guided by the theory of planned behavior, we can derive two important recommendations from our findings. (1) It is important to carefully consider the distributions of the TPB measures, making sure that ATT, SN, and PBC scores cover the whole range of the response scale; and (2) statistical analyses should include the interactions between ATT and SN on one hand, and PBC on the other.

Future research should also consider the subdimensions of ATT, SN, and PBC. The distinction between injunctive and descriptive subjective norms was taken into account in our third study, which found a significant interaction between both kinds of norm and PBC. It would also be interesting to test the ATT x PBC interaction in relation to the distinction between the two subdimensions of ATT, namely instrumental and experiential attitudes (see Fishbein & Ajzen, 2010). By the same token, it would be possible to examine the moderating role of PBC in relation to the two sub-dimensions of perceived behavioral control that have emerged in empirical research. Fishbein and Ajzen (2010) referred to these dimensions as *capacity* – the perceived ability to carry out a behavior – and *autonomy –* the extent to which performance of the behavior is viewed as being under one’s personal control. At least one study (Castanier et al., 2013) seems to suggest that these two dimensions of PBC may have different moderation effects on the ATT-intention and SN-intention relations. Future research could therefore examine the full set of interactions among the predictors of intentions: interactions between each of the two ATT factors and each of the two SN factors on one hand and each of the two PBC dimensions on the other.

Finally, we acknowledge that the three studies reported here were all conducted with Italian convenience samples, which may raise questions regarding the generalizability of our findings. Interestingly, recent work has established that, when findings are replicable, they tend to hold up across a wide range of populations and cultures (Klein et al., 2018). Nevertheless, additional studies with representative samples in other countries are needed to confirm the external validity of our findings regarding interaction effects in the context of the TPB.
